# First-Order Estimates of Coastal Bathymetry in Ilulissat and Naajarsuit Fjords, Greenland, from Remotely Sensed Iceberg Observations

**DOI:** 10.3390/rs11080935

**Published:** 2019-04-18

**Authors:** Jessica Scheick, Ellyn M. Enderlin, Emily E. Miller, Gordon Hamilton

**Affiliations:** 1 School of Earth and Climate Sciences, University of Maine, Orono, ME 04469, USA;; 2 Climate Change Institute, University of Maine, Orono, ME 04469, USA;; 3 Department of Geosciences, Boise State University, Boise, ID 83725, USA; 4 School of Marine Sciences, University of Maine, Orono, ME 04469, USA; 5 Formerly at School of Earth and Climate Sciences, University of Maine, Orono, ME 04469, USA;; 6 Formerly at Climate Change Institute, University of Maine, Orono, ME 04469, USA

**Keywords:** ice–ocean interactions, icebergs, bathymetry, optical imagery, digital elevation models

## Abstract

Warm water masses circulating at depth off the coast of Greenland play an important role in controlling rates of mass loss from the Greenland Ice Sheet through feedbacks associated with the melting of marine glacier termini. The ability of these warm waters to reach glacier termini is strongly controlled by fjord bathymetry, which was unmapped for the majority of Greenland’s fjords until recently. In response to the need for bathymetric measurements in previously uncharted areas, we developed two companion methods to infer fjord bathymetry using icebergs as depth sounders. The main premise of our methods centers around the idea that deep-drafted icebergs will become stranded in shallow water such that estimates of iceberg surface elevation can be used to infer draft, and thus water depth, under the assumption of hydrostatic equilibrium. When and where available, surface elevations of icebergs stranded on bathymetric highs were extracted from digital elevation models (DEMs) and converted to estimates of iceberg draft. To expand the spatial coverage of our inferred water depths beyond the DEM footprints, we used the DEMs to construct characteristic depth–width ratios and then inferred depths from satellite imagery-derived iceberg widths. We tested and applied the methods in two fjord systems in western Greenland with partially constrained bathymetry, Ilulissat Isfjord and Naajarsuit Fjord, to demonstrate their utility for inferring bathymetry using remote sensing datasets. Our results show that while the uncertainties associated with the methods are high (up to ±93 m), they provide critical first-order constraints on fjord bathymetry.

## 1. Introduction

Fjord bathymetry places a strong control on the ability of warm oceanic waters to enter fjords [1,2]. The presence of warm ocean water at depth in glacial fjords exerts an important influence on the dynamics of marine-terminating outlet glaciers and thus the mass balance of the Greenland Ice Sheet (e.g., [3–5]). When warm, salty, dense, subsurface Atlantic Water of subtropical origin is able to cross the Greenland continental shelf and penetrate into fjords, its heat [1,2,6–9] will enhance the submarine melting of floating ice tongues, ice mélange (a semi-rigid matrix of icebergs and sea ice), and grounded termini relative to the submarine melt rates for ice in the comparatively cool, fresh Polar Water of Arctic origin. This enhanced melting can ultimately lead to an increase in glacier mass loss through feedbacks associated with loss of frictional resistance generated at glacier termini and ice flow acceleration (e.g., [10–15]). However, despite the important influence of fjord bathymetry on glacier–ocean interactions and glacier mass balance, until recently our knowledge of fjord bathymetry was limited to a relatively small number of glaciers where observations had been acquired [16].

The resolution and spatial coverage of fjord bathymetry observations around Greenland is continually improving as the international scientific community collects new datasets (e.g., [16–20]). Multiple freely available, gridded data products now include coastal bathymetry constrained by available observations (e.g., IBCAO v3.0 [21], RTopo-2 [22], and BedMachine v3 [23]). Recent efforts have focused on removing the physical impossibilities present in many compiled datasets through the generation of realistic synthetic bathymetry and mass conservation constrained by observations (e.g., [16,24]), with a focus along the coast and at glacier termini. Each new product and version improves upon those already available as additional surveys are conducted and spatial resolution is increased.

While these efforts are critical to providing realistic topographies, particularly in ice marginal areas, the bathymetry in many of Greenland’s glacial fjords remains incompletely constrained by observations. The presence of bathymetric sills that shallow water to less than ~200 m can effectively block warm Atlantic Water located at depths of ~150–200 m from entering fjords and reaching glacier termini (e.g., [6,25,26]). Thus, mapping of sills is critical for assessing how far warm oceanic waters are able to penetrate into fjords and what impact this will have on marine-terminating glaciers [27]. However, data collected using field-based methods are time and resource intensive to obtain. Ship-based methods also rely on open water near glacier termini, which are often unnavigable due to the presence of closely packed icebergs and sea ice. Consequently, many fjords still lack the observations necessary to indicate the presence or absence of a sill.

Here we pursue a remote sensing approach that utilizes icebergs to expand upon the spatial coverage of fjord bathymetry datasets. Repeat satellite observations can be used to track iceberg motion over time. Where water depths exceed iceberg draft (i.e., keel depth), iceberg motion is controlled largely by the subsurface ocean water currents in the fjord [28,29]. In contrast, the absence of motion suggests iceberg stranding and indicates the presence of shallow waters (e.g., sills). Thus, observations of drifting and stranded icebergs can be used to shed light on fjord circulation patterns and enable the inference of qualitative (relative) bathymetry. Where iceberg drafts can be estimated, icebergs can be used to place quantitative constraints (upper or lower bounds) on water depth. Here we describe how repeat satellite observations can be used to build qualitative bathymetry maps (Sections 2.1 and 3.1). Then, we demonstrate the use of digital elevation models (DEMs) of stranded icebergs constructed from <1 m resolution stereo satellite image pairs to directly (Sections 2.2.1 and 3.2.1) and indirectly (Sections 2.2.2 and 3.2.2) infer bathymetry of shallow regions. We illustrate the utility of our methods by extending the spatial coverage of bathymetry observations in Ilulissat and Naajarsuit Fjords (Section 4).

## 2. Methods

### 2.1. Qualitative Bathymetry and Study Sites

Icebergs were identified and their general movement patterns examined for multiple fjord systems as described briefly below and in the Supplementary Material. These fjords were chosen because bathymetric measurements were available to verify the qualitative, relative bathymetry estimates inferred from observations of iceberg motion. Panchromatic and color (red, green, and blue band composite) satellite images, collected by the sensors on board the Landsat, Sentinel, MODIS, and WorldView constellations, were viewed using the LandsatLook Viewer (landsatlook.usgs.gov), GloVis (glovis.usgs.gov), Danish Meteorological Institute (DMI) satellite images (ocean.dmi.dk), and DigitalGlobe (discover.digitalglobe.com) online viewers. For each fjord, icebergs were manually identified and, wherever possible, manually tracked across multiple images as they remained in place or moved through the fjord. Iceberg size and shape were the primary features used to identify the same iceberg through time, with confirmation from visual pattern matching of surface features (e.g., snow vs. bare ice, debris).

**Figure 1.**
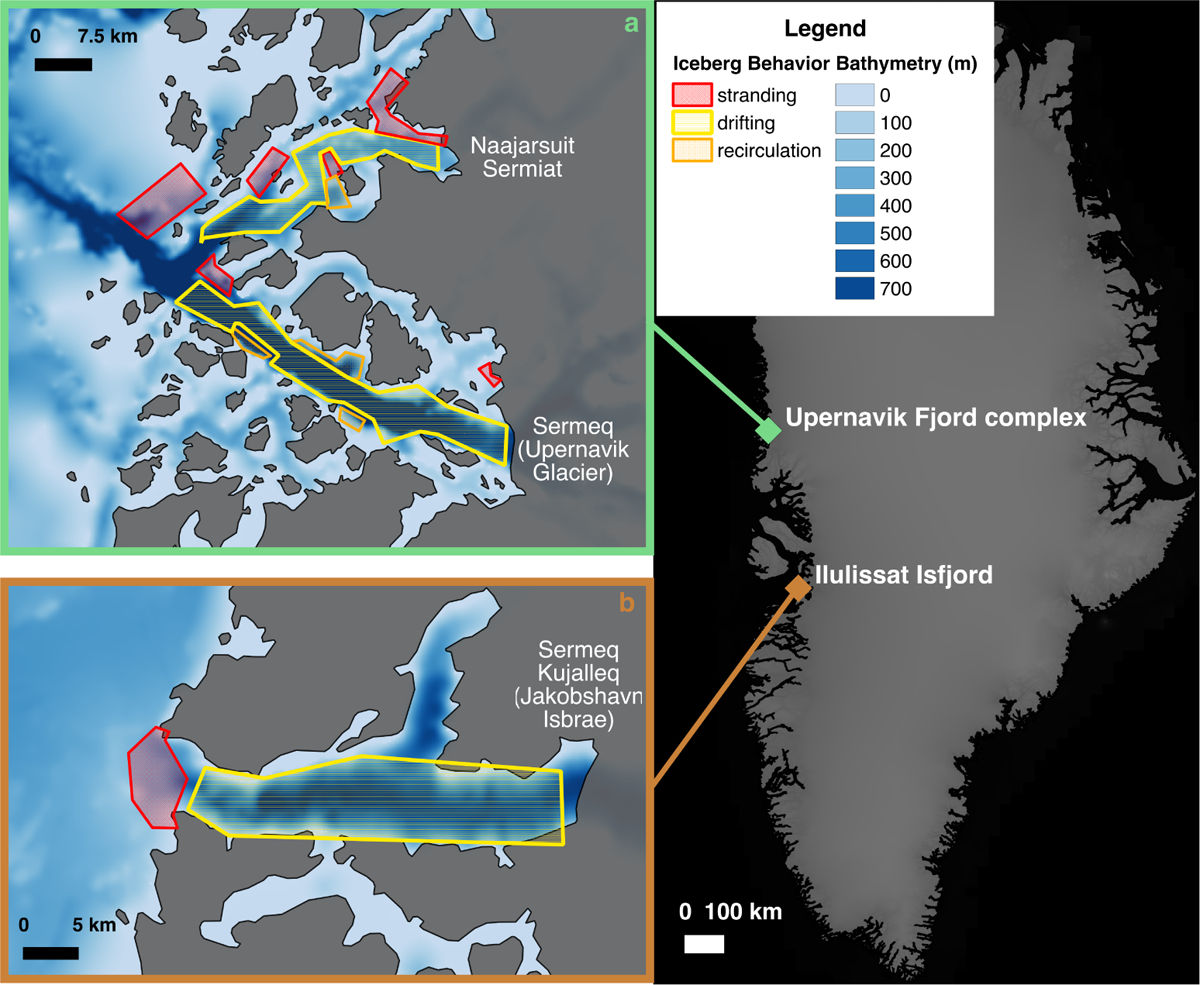
Qualitative bathymetry (i.e., relative bathymetry derived from the manual analysis of iceberg motion) overlaid on BedMachine v3 bathymetry [23] for two locations around Greenland. Areas of stranding (red) and drifting (yellow) identified by iceberg movement correspond to bathymetric highs (light blue) and lows (dark blue), respectively. Areas with no outlines were not searched. Land is shown in grey. Glaciers supplying the majority of the icebergs in each fjord are labeled, with the location of each system identified and labeled in the panel on the right.

Imagery was inspected until the operator felt confident identifying broad regions of iceberg drifting, stranding, and recirculating. We focused on images from the summer and fall, when sea ice was at a minimum and therefore did not influence iceberg mobility or detection. The number of images inspected varied depending on sea ice extent and cloud cover for a given year, but a minimum of ten images were inspected for each location. In order to be considered “stranded”, an iceberg was required to satisfy the following two criteria: (1) it occupied a semi-stationary position in numerous sequential images while other ice masses were seen freely moving around it; (2) it was free of sea ice and other ice debris to ensure that it was not simply immobilized by a matrix of floating ice (see this visually in the Supplementary Material Video S1). Regions of drifting were identified as areas that repeatedly cleared of icebergs quickly, regardless of iceberg size. Areas of recirculation were identified as locations where icebergs remained within a particular geographic area and were thus visible across multiple images but were clearly not fixed in the same location or rotating in place as a result of tidally driven fluctuations in water depth. The geographic extents of observed stranding, drifting, and recirculation areas were manually delineated as georeferenced polygons using QGIS (Figures 1 and S1) [30]).

We focused our efforts on selected regions where large outlet glaciers calve icebergs with deep enough drafts to potentially become stranded in shallow waters at or above the depth of the Atlantic Water–Polar Water interface. To minimize bias introduced by previous knowledge of a region, one operator confirmed bathymetric data were available to validate our remote sensing estimates and another operator constructed the qualitative bathymetry maps prior to viewing any bathymetry products for the area. The qualitative maps were later overlain on the measured bathymetry (Figures 1 and S1) to validate our hypothesis that iceberg drifting indicates deeper water while iceberg stranding indicates shallow water. We investigated four sites using this qualitative method; two West Greenland systems contained stranded icebergs and had sufficient WorldView stereo image pairs to estimate water depths in shallow regions (Figure 1), while two sites were not suited for this quantitative analysis (see Supplementary Material and Figure S1). In the Upernavik Fjord complex (Figure 1a) we focused our investigation on icebergs supplied by Naajarsuit Sermiat, several glacier termini (~40 km) north of Sermeq (Upernavik Glacier). We refer to the fjord into which Naajarsuit Sermiat terminates as Naajarsuit Fjord. The Upernavik/Naajarsuit region contains several shallow areas/partial sills but no distinct blocking feature across the entire fjord [31]. The second site, Ilulissat Isfjord (Figure 1b), contains icebergs solely from Sermeq Kujalleq (Jakobshavn Isbræ) and has a shallow sill extending across the entire width of the fjord where it enters Disko Bay [6,18]).

### 2.2. Quantifying Bathymetry in Regions of Iceberg Stranding

#### 2.2.1. Water Depths Derived from Freeboards

**Figure 2.**
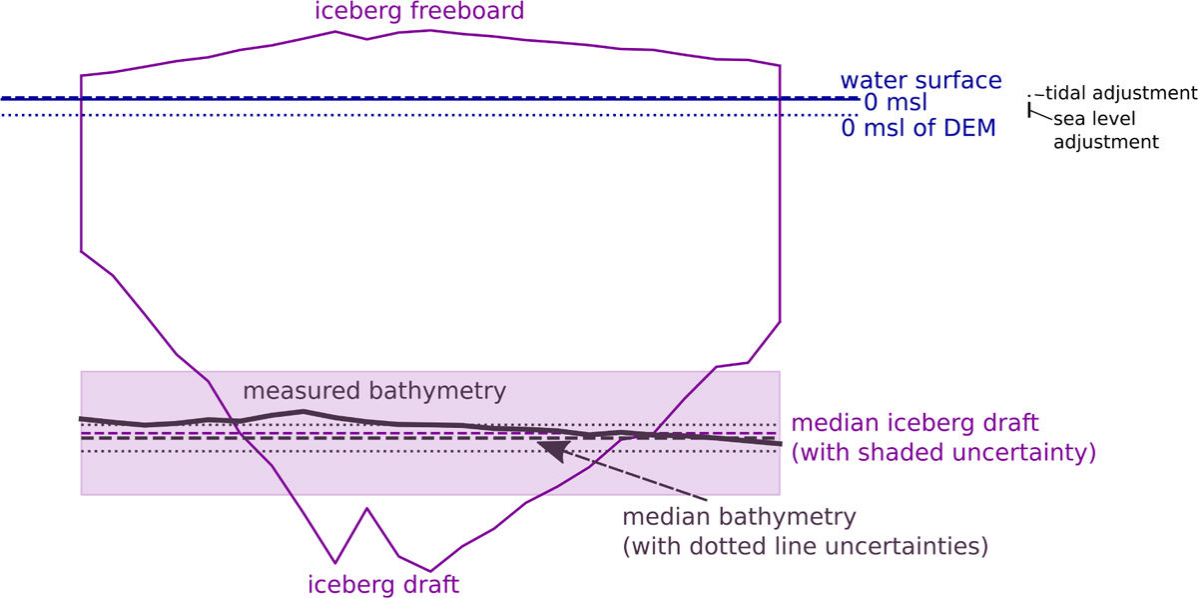
Two-dimensional iceberg schematic showing sea level and tidal adjustments, assumed iceberg shape, and bathymetry. Values shown are based on a transect across a stranded iceberg from 10 June 2014. A sea level (blue dotted line) adjustment was applied to each DEM to vertically coregister it such that open water pixels were 0 m. A tidal adjustment (blue dashed line) applied to each iceberg draft estimate vertically coregistered all of the water depth estimates to 0 m msl. Iceberg freeboard was extracted from the DEMs and used to infer iceberg draft (purple line) on a pixel-by-pixel basis. Iceberg walls were assumed to be vertical. Median iceberg draft (and thus water depth) and associated uncertainties are shown by the purple dashed line and shading, respectively. The gradual nature of the measured bathymetry (solid brown line), including the median value (dashed brown line) and associated median absolute deviation (MAD) uncertainties (dotted brown line), are also shown.

The premise of our approach is that the draft of stranded icebergs grounded on bathymetric highs can be used to infer the water depth in each iceberg’s location. Iceberg draft depends on the iceberg’s shape [32,33], which is non-unique, and ratios of iceberg draft to width vary [32,34–36], making draft difficult to infer solely from measurements of surface dimensions. Here we infer iceberg drafts from freeboard observations assuming simplified submerged geometries (Figure 2) as described below.

Based on the results of the qualitative analysis, stranded icebergs were identified in WorldView stereo image pairs for two regions: Ilulissat Isfjord and Naajarsuit Fjord. These sub-meter resolution WorldView stereo satellite images were then used to construct ~2 m horizontal resolution DEMs with NASA’s Ames Stereo Pipeline (ASP) software package [37]. To reduce computation time and resource requirements, DEMs were constructed for scene subsets containing only the stranded icebergs using ASP’s stereo_gui command. Then, each DEM was adjusted to local sea level following the methods of Enderlin and Hamilton [38] (Figure 2). Specifically, a small subset of open water pixels near each iceberg was used to vertically shift the entire DEM so that open water was at an average elevation of 0 m. This adjustment inherently removes any potential DEM bias due to orbital uncertainty as well as offsets associated with the tidal height at the time of image acquisition relative to mean sea level (msl).

Each stranded iceberg was manually outlined in its respective DEM and the iceberg surface elevation with respect to water level (i.e., freeboard) was extracted for each DEM pixel within the iceberg outline. We assumed pixel-by-pixel hydrostatic equilibrium such that each iceberg had vertical sides and the submerged bottom surface was an exaggerated reflection of freeboard (Figure 2). Iceberg draft, *d*, was estimated from the freeboard observations, *h*, as

(1) $$d=h\left(\frac{\rho_{i}}{\rho_{w}-\rho_{i}}\right),$$

where *ρ*_*i*_ and *ρ*_*w*_ are the fjord-specific densities of ice and ocean water, respectively. The fjord-specific density values used in our calculations are given below. In order to vertically coregister each iceberg draft relative to 0 m msl of the local geoid and obtain the actual water depth at the time of observation, the modeled tidal height at the time of the DEM image pair acquisition was applied to each draft estimate (Figure 2). Tidal heights are from the Arctic Ocean Tide Inverse Model (AOTIM-5) [39] for a site near each fjord’s mouth, as in Enderlin and Hamilton [38]. Iceberg drafts, and thus water depths, were estimated using this approach for a total of 27 (10) stranded icebergs using seven (two) stereo image pairs for Ilulissat Isfjord (Naajarsuit Fjord).

The requirements and challenges of creating DEMs using ASP are discussed in more detail in Enderlin and Hamilton [38] and Shean et al. [37]. The presence of large areas of open water around icebergs poses a challenge to the pattern matching employed by ASP, often resulting in DEMs with large areas of no data and spurious heights of tens of meters for some open water pixels. Each stranded iceberg DEM was inspected to confirm that enough of the iceberg was successfully mapped and the quality of the DEM was high enough to provide a representative range of freeboard values as well as accurate sea level adjustments (typically 1–5 m after the influence of tidal height is removed). Areas used for sea level adjustment were carefully selected to avoid inclusion of spurious open water pixels. Inclusion of these pixels otherwise resulted in unrealistic sea level adjustments (>10 m), consequently providing poor estimates of water depth.

To verify the method, bathymetry estimates derived using the freeboard method were compared with gridded datasets of sonar-derived bathymetry. The bathymetry measurements used for the comparison were collected using multibeam echosounding and gridded to 20 m by 20 m and 25 m by 25 m horizontal resolution, for Ilulissat and Naajarsuit Fjords, respectively ([18,31], respectively). Given that the shape of the submerged portion of the iceberg cannot be inferred from surface observations, this comparison allowed us to determine the most representative iceberg draft value (e.g., mean, median, maximum) for inferring water depths at stranding locations. To develop these metrics, each stranded iceberg outline was overlaid on the corresponding bathymetric grid and the water depth at each covered gridpoint extracted. The horizontal uncertainty in ASP produced DEMs using WorldView images is <3–5 m [37], which is close to the pixel size of the DEMs used for the analysis (2 m by 2 m) and much smaller than the horizontal extents of the icebergs (minimum iceberg width of all icebergs delineated was 71 m, with a median width of 336 m). Thus, the georeferencing accuracy of our iceberg polygons is well under the grid spacing of the measured datasets, making it unlikely that any gridded points were included/excluded as a result of georeferencing errors. Since there is no physical reason why the water depths extracted from the gridded bathymetric datasets should follow a normal distribution, we use the median, rather than the mean, of the sonar-measured bathymetry values to represent the “true” water depth for each iceberg’s location.

#### 2.2.2. Water Depths from Depth–Width Ratios

A particular challenge of the freeboard method of estimating water depth lies in its dependence on the temporal overlap between iceberg stranding and the collection of WorldView stereo image pairs. This is especially problematic in regions where the bathymetry and/or iceberg drafts are such that there is not a perpetual abundance of stranded icebergs. However, even in locations with a continual fleet of stranded icebergs (e.g., Ilulissat Isfjord), the number of bathymetry points derived using this method is limited by the availability of high-resolution freeboard observations derived from cloud-free WorldView stereo image pairs. To overcome this limitation and increase the spatial coverage of our inferred bathymetry dataset, we used the available iceberg DEMs to derive fjord-specific iceberg depth–width ratios. Then, we applied these ratios to the measured widths of stranded icebergs from Landsat 8 and Sentinel-2 panchromatic images to infer water depths.

**Figure 3.**
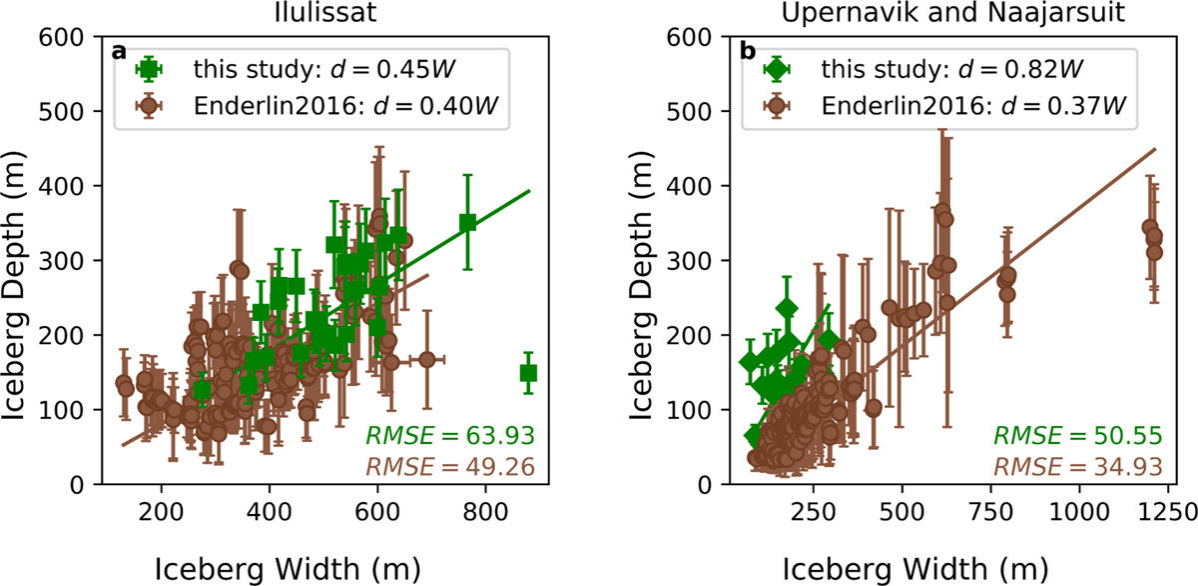
Depth–width ratios in Ilulissat and Naajarsuit Fjords. Green squares/diamonds show the results from this study, while brown circles show results from Enderlin2016. Best fit lines are used to determine the depth–width ratio, with RMSE values as shown and statistically significant *p* values (<0.05) for all fits. (**a**) Results in Ilulissat Isfjord. (**b**) Results from the Upernavik region, where the icebergs comprising the two datasets were derived from different parent glaciers.

We derived depth–width ratios for each fjord using iceberg width and median iceberg depth. Iceberg width was taken as the minor axis of a minimum bounding ellipse fit to each iceberg polygon. In Ilulissat Isfjord, the depth–width relationship was derived using the 27 stranded iceberg DEMs (Figure 3a, green squares). In Naajarsuit Fjord, to supplement the small number (ten) of stranded iceberg DEMs available, additional DEMs of eight non-stranded icebergs were constructed and included in establishing the depth–width relationship (Figure 3b, green diamonds). The ratios were calculated as the slope of a best fit line with a forced intercept of (0, 0) [36] and are statistically significant with *p* values < 0.05. To check the robustness of our ratios given the sparseness of our datasets, we also computed depth–width relationships for the much larger DEM-derived iceberg datasets used to establish iceberg melt rates in Enderlin et al. ([36], hereafter referred to as Enderlin2016) (Figure 3, brown circles). Their data were extracted from DEMs and provided as median drafts and total planar areas for each iceberg. Planar area was assumed to represent a circular iceberg and used to calculate iceberg width as two times the radius of a circle covering that area. Then, this width was compared to the median draft for each iceberg, where draft was derived as in this study using the assumption of hydrostatic equilibrium for iceberg freeboards extracted from a DEM. We identified and manually outlined a total of 50 (Ilulissat) and 34 (Naajarsuit) stranded icebergs using Landsat 8 and Sentinel-2 imagery, calculated their widths, and then applied the fjord-specific depth–width relationship to estimate water depth.

#### 2.2.3. Error Analysis

The bathymetry derived from iceberg freeboards is subject to a number of sources of uncertainty. These can be broadly categorized as errors stemming from the vertical accuracy of the DEM and errors that result from the assumptions made in employing the method to derive water depth estimates. Systematic bias in iceberg freeboard due to uncertainty in the satellite position is effectively removed during the local adjustment of open water pixels near each stranded iceberg. After accounting for the vertical adjustment due to tidal height using AOTIM-5, we found that the bias adjustments on iceberg DEMs ranged from 0.03–9.9 m and varied systematically by DEM. The largest mean residual for any of the DEMs was <3 m, with typical mean residual values of 0.5–1 m for a given DEM. Random errors due to mis-matching of pixels in the stereo images are reduced by ASP through erosion and mean difference to neighbors filtering applied to the pixel disparity map prior to point cloud generation (triangulation) [37]. Both Enderlin and Hamilton [38] and Shean et al. [37] estimated random uncertainty of vertically coregistered DEMs to be ~2–3 m.

**Figure 4.**
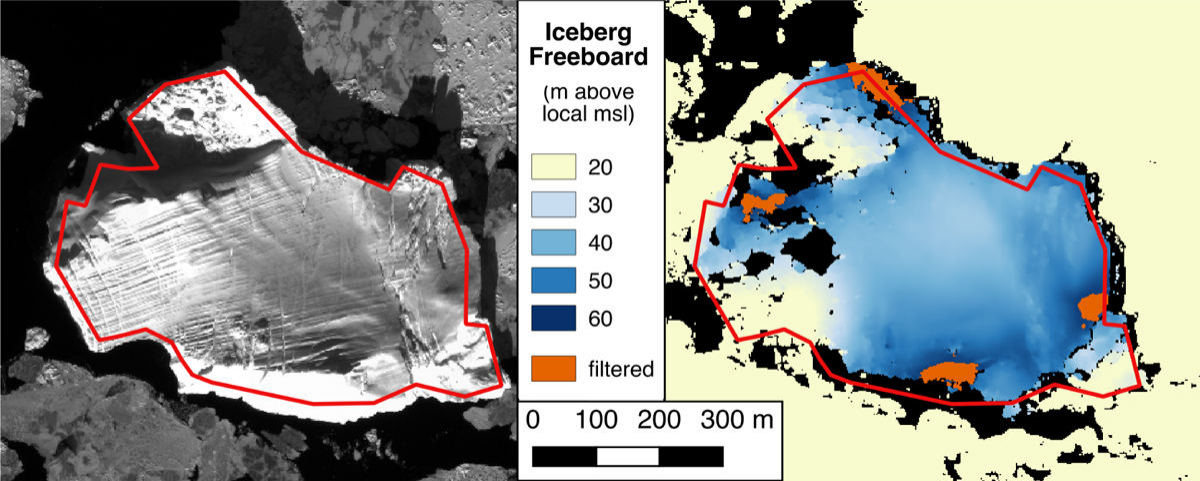
Maximum iceberg freeboards in DEMs. A panchromatic image (left panel, imagery© 2015 DigitalGlobe, Inc.) and DEM (right panel) of an iceberg stranded in Ilulissat Isfjord (image pair acquired 16 March 2015) show maximum iceberg freeboards tend to occur over and along the boundaries of highly reflective and no data regions. The iceberg is outlined (red) in both panels. In the panel showing the DEM, black indicates no data portions of the DEM while orange indicates high values filtered out by the three MAD filter. The freeboard elevations have been limited to a portion of their full range to highlight the maximum values.

Despite the automatic filtering done by ASP to minimize vertical errors, during manual inspection of the final DEMs we observed anomalous maxima values over or along the boundaries of portions of the input images that are highly reflective (Figure 4). These anomalously high freeboard values generally bordered no data portions of the DEM. Inspection of the good pixel map produced for the DEM sometimes identified these local maxima pixels as bad. To ensure that our draft estimates were not skewed by these “blunders” in pixel matching, we applied a three median absolute deviation (MAD) filter to the range of draft values for each iceberg. Manual inspection of the DEMs before and after application of the filter indicates that this simple filtering approach is effective, removing the majority of blunders while preserving the full range of more accurate elevations (Figure 4).

To determine the uncertainty on our bathymetry estimates (Figure 2), we propagated uncertainties in densities and freeboard through our draft calculations using standard error propagation techniques. Ice and ocean density vary spatially and temporally, and local measurements for these parameters are not available in all fjords around Greenland. The density of pure glacial ice is typically taken to be 917 kg/m^3^. Filling in of void space with meltwater would increase this value, while increased iceberg fracturing and the presence of snow and firn would effectively lower it. The presence of large quantities of entrained debris will also influence the iceberg density, but this influence is not well constrained and likely has a minimal impact on the density of our icebergs for two reasons: the icebergs in our study sites visually appear to contain little sediment, and the bulk of any sediment that they initially contained was probably dropped over the days to months since the icebergs calved [40]. We assume that the icebergs do not contain any firn as they have calved from glaciers with bare ice exposed at their termini. Given the unquantifiable unknowns in iceberg density and following previous investigations, we used an iceberg density of 900 ± 20 kg/m^3^ [38,41–43], which assumes a small amount of void space relative to solid glacial ice and accounts for the unknown differences in ice density between icebergs due to differences in void space, fractures, composition, and refreezing. This results in iceberg density contributing the largest component of uncertainty to our draft/depth estimates. Ocean water density varies by location and depth and with time. Fjord-specific measured near-surface ocean densities plus or minus two sigma error (i.e., two standard deviations) of 1027.3 ± 1.0 kg/m^3^ [44] and 1028.5 ± 1.0 kg/m^3^ [45] were used for Ilulissat Isfjord and Naajarsuit Fjord, respectively. Although ocean water density likely does not remain constant across all of the stranded icebergs, the uncertainty in our depth estimates due to ocean water density is an order of magnitude smaller than that due to uncertainties in iceberg density. Given its relatively small contribution to water depth uncertainty and the lack of sufficiently highly temporally and spatially resolved ocean water density observations to extract water density estimates for each iceberg, we use these fjord-specific values throughout our analysis.

An important component of uncertainty in iceberg draft values stems from the influence of stranding on the validity of the assumption of hydrostatic equilibrium. The observed freeboard of a stranded iceberg may be influenced by iceberg ploughing, tilting, and/or tides. Where an iceberg has scoured into the sediment, the freeboard may be artificially decreased and the assumption of hydrostatic equilibrium then results in an underestimation of water depth. Paleo and modern iceberg scours measured on the U.S. Atlantic coast, Argentine margin, central North Sea, and North Falkland Basin can reach up to 10–20 m deep (e.g., [46–49], respectively). However, since most scours average only a few meters in depth (e.g., [50]), we assume that the potential effects of scouring on our inferred depths are within our uncertainty estimates. Similarly, and potentially concurrently, iceberg stranding may cause the subaerially exposed portion of the iceberg to tilt relative to flotation, changing the observed freeboard. A lack of observations precludes quantification of this uncertainty component, but it is likely to be minimal relative to other sources of uncertainty given the small (~2–3 m) tidal ranges for our study sites. The easiest component of vertical uncertainty on our freeboard to measure is that resulting from the height of the tide at the time of image acquisition. For instance, where an iceberg stranded at high tide has its freeboard measured at low tide, the freeboard will be exaggerated relative to its value were the iceberg floating, biasing our water depth estimates.

In order to quantify the potential uncertainties stemming from deviations from hydrostatic equilibrium, we compared inferred water depths for the same iceberg stranded in Ilulissat Isfjord across two of our DEM dates (16 March and 25 April 2015). These data indicate that the water depth uncertainty introduced by deviations from hydrostatic equilibrium is on the order of 10 m (inferred water depths were 324 m and 313 m, at tidal heights of −0.64 m and −0.07 m, respectively). A portion of this difference is likely due to mass loss during the ~5.5 weeks between acquisition dates, suggesting that the potential water depth biases introduced by deviations from hydrostatic equilibrium are likely <10 m. Since we do not have repeat DEMs of all stranded icebergs, we calculated the tidal stage component of freeboard uncertainty for each iceberg using modeled tidal heights. Specifically, we determined upper and lower uncertainty bounds on freeboard equal to the difference between the modeled tidal height at the time of image acquisition and the nearest local maximum and minimum, respectively. Freeboard uncertainties ranged from 0.35 to 1.21 m (median: 0.72 m). Standard error propagation of these density and freeboard uncertainties ultimately provided constraints on our water depth (i.e., bathymetry) estimates, with uncertainties ranging from 10 to 63 m (median: 34 m). Because the magnitude of the component uncertainties varies across fjords and time, errors were calculated individually for each bathymetric estimate.

Vertical uncertainties on bathymetry estimates derived using the depth–width method stem from the same sources as the vertical uncertainties described above for the freeboard method, including unquantifiable uncertainties in the submerged iceberg shape, and the propagation of uncertainties in iceberg width. Since the vertical uncertainties stemming from tidal height are asymmetric, we provide conservative uncertainties by assigning the maximum magnitude vertical uncertainty to each iceberg DEM used in establishing the depth–width ratio. The median of these individual vertical uncertainties provides overall water depth uncertainties of 42 m and 26 m (Ilulissat and Naajarsuit Fjords, respectively) for values derived using the depth–width ratio. Uncertainties in iceberg widths stem from operator bias and image resolution and influence the vertical errors associated with inferring water depths. Errors resulting from image resolution are subjective because there is no way to determine the true iceberg width within the pixel resolution. For icebergs outlined in WorldView images and/or DEMs (pixel resolution ≤2 m by 2 m), operators outlined icebergs conservatively to ensure all pixels within the outline were within the iceberg margins. For icebergs outlined in Landsat and Sentinel images (pixel resolution of 15 m by 15 m and 10 m by 10 m, respectively), the larger pixel size resulted in abundant mixed ice–water border pixels around each iceberg. Operators outlined icebergs assuming that the actual iceberg edge was towards the iceberg center relative to this zone of mixed pixels. To quantify operator bias, all operators outlined the same two icebergs in Ilulissat Isfjord at several points in time throughout the data collection period. Widths ranged from 578–612 m and 384–441 m for the two icebergs, respectively. Applying our depth–width ratios to the range of the widths (34 and 57 m) translates to vertical uncertainties of ≤26 m. Since the operator bias in determining iceberg widths results in a vertical uncertainty component less than that stemming from the establishment of the depth–width ratio, we use the larger of the vertical uncertainties on our depth–width derived water depths (42 m and 26 m for Ilulissat and Naajarsuit Fjords). As noted above for the freeboard method, image georeferencing accuracy is not relevant given the georeferencing accuracy of the imagery relative to the iceberg size and gridded dataset resolution.

## 3. Results and Evaluation of Methods

### 3.1. Qualitative Bathymetry

Figures 1 and S1 show the results of our qualitative examination of relative bathymetry inferred from iceberg movement overlaid on the BedMachine v3 bathymetry product [23]. The BedMachine output is forced by observations in the areas shown, with errors close to 0 m for most parts of the fjords and larger errors (>150 m) near glacier termini and land boundaries where observational coverage is limited (not shown). Thus, BedMachine provides reasonably accurate bathymetry for assessing our qualitative method in these fjords. Because this is a qualitative method, errors cannot be quantified; however, the overlaid maps indicate a good agreement between relative bathymetry as suggested by our method and the actual relationships established by measured datasets (Figures 1 and S1).

Examination of the overlays suggests that regions of stranding and drifting correspond with relative bathymetric highs and lows (i.e., shallower and deeper water), respectively (Figures 1 and S1). In basins without measured bathymetry for confirmation, deeper regions inferred by rapid iceberg transport may indicate the presence of deep troughs that channel subsurface water on the shelf towards the glacier terminus. An investigation of relative iceberg drifting speeds and pathways, while beyond the scope of this study, may provide additional insight into the fjord’s bathymetric shape in these locations. Regions where icebergs were observed to recirculate without becoming stranded indicate areas with a more complicated bathymetry and tend to occur proximal to land features, particularly those associated with non-linear fjord geometries.

### 3.2. Quantitative Bathymetry

#### 3.2.1. Freeboard Method

**Figure 5.**
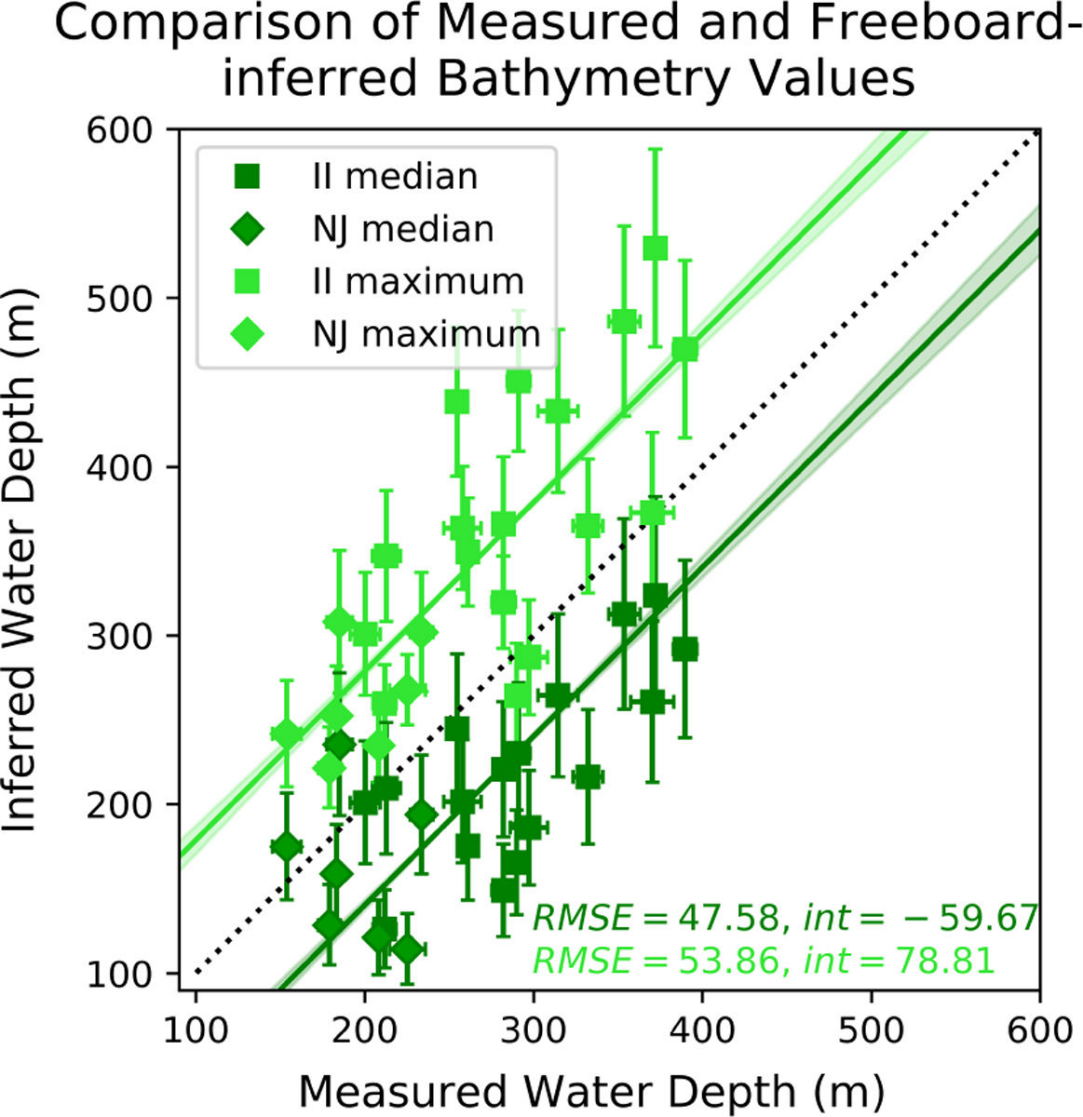
Measured and freeboard-inferred water depths for stranded icebergs in Ilulissat and Naajarsuit Fjords. Depths are in meters relative to 0 msl of the local geoid. Inferred depths are derived using the freeboard method, as described in the text. The light green (dark green) points compare the maximum (median) inferred and measured values. The intercept (int) and RMSE value for the best fit lines (forced slope of one) are shown in the corresponding color. Squares (diamonds) correspond to icebergs stranded in Ilulissat Isfjord-II (Naajarsuit Fjord-NJ). The black dotted line shows a 1–1 relationship.

Water depths taken from gridded bathymetry datasets are compared to those derived from iceberg freeboards in both study regions (Figure 5). The freeboard-inferred median (maximum) draft tends to under- (over-)estimate the sonar-measured water depth. This result makes sense when the complex submerged geometry of icebergs is considered. Icebergs are unlikely to have smooth, level bottoms. Thus, the iceberg will likely become stranded where its draft exceeds the median. It is also unlikely that the iceberg’s mass is perfectly distributed below the surface such that each freeboard elevation is exactly balanced by a proportional mass directly beneath it, as is suggested by the assumption of hydrostatic equilibrium on a pixel-by-pixel basis. Thus, drafts inferred from freeboard maxima may exceed the true maximum iceberg draft, resulting in an overestimation of water depth. Taken together, the data suggest that the median and maximum values can be used to place bounds on the bathymetry for a particular location.

The MAD provides the uncertainty for sonar-derived water depths, ranging from 0.6 m to 12.8 m (median 2.8% of the measured water depth). Error for each inferred water depth is propagated as described in Section 2.2, and uncertainties range from 10 m to 63 m (median 18% of the inferred water depth). The use of median and maximum water depth values inherently captures propagated variations in iceberg freeboard. Thus, we suggest that propagated tidal uncertainties, rather than freeboard MAD values, provide a more appropriate measure of bathymetric uncertainty.

The median and maximum inferred water depths provide important constraints on actual water depth in a given location. However, most applications (e.g., gravimetry processing inputs, circulation models, and bathymetric maps) require input of a single water depth value for each location rather than a range of possible values. To assess whether there is a more representative metric to approximate water depth, we constructed a cumulative distribution function (CDF) for each iceberg’s pixel-by-pixel draft estimates and then found the percentile at which the CDF intersected the median sonar-derived depth. For Ilulissat (Naajarsuit) Fjord, percentiles ranged from 50%–100% (14%–93%), where the 100th percentile indicates that the median sonar-derived water depth was greater than the maximum inferred water depth. In both locations, we found that 82% of pixel drafts were shallower than the median sonar-derived water depth. To test the utility of this representative value for inferring a single water depth, we extracted the 82nd percentile inferred water depth from each CDF. Then, we compared this inferred water depth to the median measured water depth (not shown). Although this approach produced reasonable water depths, we completed our analyses using median inferred values because of the broad range of matched percentiles for any given iceberg (14%–100%) and the lack of compelling physical rationale for the similar median percentile values in the two fjords.

#### 3.2.2. Depth–Width Ratio Method

The depth–width relationships established in this study are presented along with previously published median ratios in Table 1. A comparison of the values presented suggests our depth–width ratios are reasonable and represent stable iceberg geometries. Small differences in the depth–width ratio for Ilulissat Isfjord derived from data in this study and Enderlin2016 are likely driven by differences in assumptions about iceberg shape and stranding of our icebergs. The use of best fit ellipses will tend to overestimate iceberg width relative to a circle, in turn causing a decrease in depth–width ratio. This would tend to drive the ratios closer together. Thus, the differences in depth–width ratios presented herein are minimized with respect to our assumed iceberg shapes, suggesting that the observed differences are driven by other factors. Because they are grounded on a bathymetric feature and cannot remain floating throughout the tidal cycle, stranded icebergs may have artificially high freeboards relative to floating icebergs with the same width. This overestimation of freeboard would result in too-large draft estimates, in turn raising the depth–width ratio. In regions where the iceberg is resting on soft sediments, this effect may be compensated by the formation of pits [49]. This lowering of the iceberg into the sediments at lower water levels could result in too-small draft estimates, decreasing the depth–width ratio. However, in the shallowest regions of Ilulissat Isfjord where icebergs become stranded, the sediment layer is thin enough [18] that this effect is unlikely to measurably impact our draft estimates.

The stranded nature of the icebergs will result in different proportional rates of mass loss relative to floating icebergs, resulting in different depth–width ratios. Specifically, water shear and wave action along the iceberg’s lateral margins will tend to promote the formation of waterline notches and subsequent calving (e.g., [51–53]), reducing iceberg width and driving an increase in an iceberg’s depth–width ratio. Simultaneously, its contact with the bed will serve to stabilize the iceberg and reduce the likelihood of overturning even as the depth–width ratio increases [54]. Because rates of relative water shear are likely to be higher for stranded icebergs relative to floating ones, we would expect to see higher depth–width ratios for stranded icebergs. In addition, the stranded icebergs have had longer to decay relative to the icebergs floating within the fjord, which could also drive a change in iceberg geometry and result in different depth–width ratios with distance from the calving front. Thus, we suggest that while sample size likely plays an indeterminate role, the primary cause of our higher depth–width ratio in Ilulissat Isfjord relative to that calculated using the Enderlin2016 dataset is driven by the stranded nature and older age of our icebergs and the associated differences in iceberg shape. The much larger difference between the two ratio values in the Upernavik region is likely the result of differences in the calving processes of the source glaciers (Sermeq and Naajarsuit Sermiat), though our value’s similarity to that calculated by Hotzel and Miller [32] suggests it is within the expected range of iceberg depth–width ratios. Among the other studies of Arctic icebergs and their size characteristics (e.g., [34,55]) we were unable to find additional published median depth–width (or height–width) ratios with which to compare our data, though El-Tahan and El-Tahan [55] provided potential upper and lower bounds for establishing a depth–width relationship.

#### 3.2.3. Combining Quantitative Methods

**Figure 6.**
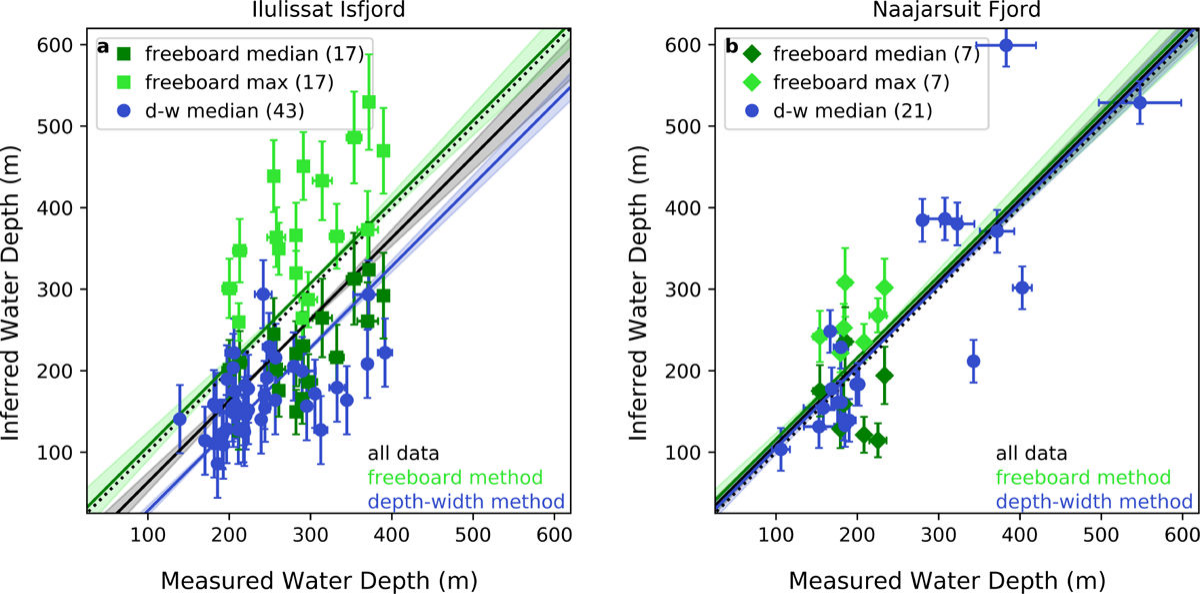
Comparisons of measured and inferred water depths for stranded icebergs using both remote sensing methods. Depths are in meters relative to 0 msl of the local geoid. Inferred depths are derived using the freeboard (squares/diamonds) and depth–width ratio (circles) methods. (**a**) Results from Ilulissat Isfjord. (**b**) Results from Naajarsuit Fjord. The black dotted line shows a 1–1 relationship. Black, green, and blue solid lines show best fit lines (forced slope of one) to the data (the methods combined (all symbols), the freeboard method (squares/diamonds), and the depth–width ratio method (circles), respectively) with shaded 95% confidence intervals. The RMSEs and intercepts of the best fit lines are shown in Table 2 in corresponding colors. The number of stranded icebergs used for each method is shown in parentheses in the legend.

In agreement with the freeboard method, the median water depths estimated by the depth–width method tend to fall below the 1–1 line (Figure 6). This result is unsurprising given the depth–width relationships are derived using median iceberg draft values. To evaluate our methodology and provide conservative uncertainties for the methods overall (rather than for each depth estimate), we calculate RMSE values for our residuals relative to multiple trendlines with a forced slope of one (Table 2). We use RMSE values, rather than R^2^ values, because the latter is highly sensitive to the number of observations and is thus not necessarily a reliable indicator of the methods’ effectiveness for a small sample size. In this case, the RMSE provides a more intuitive metric for evaluating the accuracy of inferred bathymetry in locations lacking direct observations. RMSE values computed for each method relative to the 1–1 line (slope = 1 and intercept = 0, bottom portion of Table 2) indicate the overall performance of the methods. Because in some cases the data is biased depending on the use of median or maximum values (i.e., almost all of the data points lie above or below the 1–1 line), we also computed the intercept of a fit trendline with a forced slope of one for each method (blue and green lines in Figure 6) and combined (black lines in Figure 6). The fit intercepts then provide a quantification of the bias in under- or over-estimating inferred depth values.

A comparison of RMSE values and their associated bias estimates indicate the overall uncertainty of water depths derived using these methods. Even where bias estimates are large (intercept = −72), RMSE values suggest that overall the methods can be used to infer water depths to within ±93 m of measured water depths. This conclusion holds despite unquantifiable uncertainties in iceberg mass distribution not reflected in the error bars on individual points, suggesting that our methods place reasonable quantitative bounds on actual water depth. Combining the methods enables us to take advantage of their individual strengths. First we use the freeboard method to infer as many water depths as possible and establish a fjord-specific depth–width relationship. Then we employ the depth–width method, which requires significantly less person hours and computing power to derive each water depth estimate, to capture the full spatial extent of shallow regions.

### 3.3. Evaluation

Although crude, the qualitative observations presented here (Figures 1 and S1) provide a robust first-order approximation of bathymetry in basins with few or no bathymetric measurements at little to no cost. This information is helpful for: (1) providing context for point and centerline datasets, where a few high bathymetric points may be interpreted as outliers rather than detections of key features; (2) identifying the presence and probable extent of large features such as sills; (3) prioritizing locations for in situ measurements by ship or aircraft.

A comparison of the numerical water depths inferred in Ilulissat and Naajarsuit Fjords (Figure 6) yields several important insights. First, the establishment of a fjord specific depth–width ratio is critical to the success of inferring water depths from stranded icebergs for which only widths are available (i.e., non-stereo images). Whether or not this relationship can successfully be inferred based on known features of the parent glacier, such as ice thickness at the terminus, rather than through the generation of multiple iceberg DEMs, is beyond the scope of this study but presents an interesting avenue for future investigation. Second, although a range of iceberg sizes is preferred, even a relatively small (18 icebergs) dataset can be used to establish a depth–width relationship that will produce reasonable water depth estimates (<73 m uncertainty) for stranded icebergs across a much broader range of iceberg sizes. Third, using remote sensing data to infer bathymetry, and in particular quantify water depth, may be a method best suited for application to regions with a high abundance of stranded icebergs (e.g., regions with sills blocking the path of all large icebergs from exiting the fjord), because this increases the likelihood of the presence of a large number of stranded icebergs that are fairly well spatially distributed and visible in multiple imagery sources.

The water depths derived using both methods illustrate that we can use these remote sensing-based methods to estimate water depths within ±93 m of measured water depth, with the typical uncertainties less than this amount (median iceberg draft uncertainty: 34 m). The linear fits to the data suggest that the methods estimate water depth accurately enough to provide useful information about the local water depth of previously uncharted sills and shallow regions, despite the uncertainties stemming from unknown submerged iceberg shape, influence of the iceberg-bed interactions, and iceberg density. Although these uncertainties are likely too large to use the methods to determine the ability of Atlantic Water masses to reach an individual glacier terminus, they still provide a useful metric for indicating regions where sills are present and further observations are needed to better constrain bathymetry.

## 4. Applications: Deriving Bathymetry in Unmapped Regions

**Figure 7.**
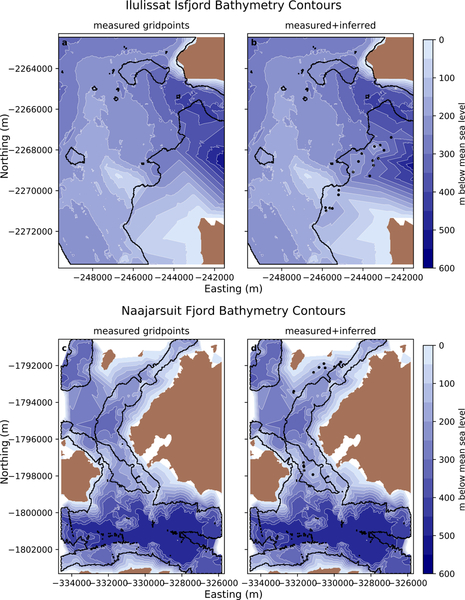
Bathymetric contours showing the utility of remote sensing inferred water depths in unmeasured areas. The top and bottom rows show the results from Ilulissat Isfjord and Naajarsuit Fjord, respectively. The left panels (**a,c**) illustrate contour lines (white, 50 m) using only multibeam observations. The right panels (**b,d**) show the improvements made by including our water depth estimates in portions of fjords where no observations exist. The black outline shows the extent of the measured datasets, where interior outlines within the outermost extent indicate holes in coverage (showing individual gridpoints would obscure the contours). Black circles indicate the location of remotely sensed data points added in the panels on the right.

To illustrate the utility of our methods, we used them to obtain bathymetry estimates in several fjords. Specifically, we applied our methods to extend observational coverage in two regions. We illustrate the outcome by combining the sonar-derived observations with our inferred bathymetry estimates, linearly interpolating the combined dataset onto a regular grid that matches the original sonar-derived observation grid spacing, and contouring the results (Figure 7).

In Ilulissat Isfjord (Figure 7a,b) the extension of the dataset farther into the fjord clearly improves the contouring, effectively illustrating the presence of shallow portions of the known sill not readily visible in the observations from Schumann et al. [18]. At the southern portion of the fjord entrance into Disko Bay, there is a lack of measurements extending from the south-central (relative to the extent of our figure) shallow region to the peninsula that comprises the northeastern most land tip here. Schumann et al. [18] suggested that this bathymetric high is a continuation of the land tip, which is supported by our extension of observational coverage. Further observations are needed, however, to fully resolve this feature.

In Naajarsuit Fjord (Figure 7c,d), the deepest portions of the fjord are well mapped. However, water depths inferred using remote sensing in the shallow regions between the measured transects provides added detail on the nature of shallowing towards small islands situated within the fjord. This is illustrated well in the northern extent of Figure 7c,d, where four stranded icebergs indicate water depths in excess of those derived from interpolation between sonar-based measurements and land.

Water depths inferred using these remote sensing methods provide important constraints on water depth in shallow regions where no measurements are available. Applying the methods to quantify water depths requires deep-drafted icebergs, relatively shallow waters, and sufficient satellite imagery to both detect iceberg stranding and construct iceberg DEMs. Many suitable areas for application of the methods have recently been mapped as observational coverage around Greenland has expanded in the last several years [19]. The techniques described herein provide a means to expand the spatial coverage of bathymetry maps, including in regions where glaciers are retreating beyond the coverage of ice penetrating radar-derived glacier bed topography maps. Many of Greenland’s marine-terminating outlet glaciers currently have termini resting on shallow pinning points, including sills (e.g., [13,16,27]). As these glaciers retreat, their termini may calve large, full thickness icebergs that will become stranded on the now-exposed sills. The methods demonstrated herein can be used to estimate the height of these sills, enabling more accurate predictions of the future presence and impacts of Atlantic Water masses on continued glacier evolution without the need for continual ship-based remapping of bathymetry at glacier termini.

## 5. Conclusions

Warm ocean waters circulating off the coast of Greenland have the potential to drive significant ice mass loss from the continent through their interactions with the ice sheet’s marine-terminating outlet glaciers. The presence and movement of these warm waters at depth in glacial fjords are topographically steered by the bathymetric features between the shelf and glacier termini. However, despite recent advances in the number of observations and spatial resolution of bathymetric datasets (e.g., BedMachine, RTopo, IBCAO), changes in glacier termini positions and the high resource intensity of ship- and air-based bathymetric data collection methods means that bathymetry in many of Greenland’s fjords will remain unmapped.

The central premise of our remotely sensed iceberg-based bathymetry mapping methods stems from the fact that full thickness icebergs calved from many of Greenland’s large outlet glaciers have drafts that exceed the water depth of shallow regions located within the fjords into which they calve. Thus, icebergs that can be identified as stranded on bathymetric highs can be used to qualitatively infer the presence of shallow regions and sills. In order to quantify water depths in these shallow regions, we used two related methods. DEMs of icebergs produced from sub-meter resolution stereo image pairs were used to convert observations of iceberg freeboard to iceberg draft estimates. Because the icebergs were stranded, the inferred draft values were used to constrain water depths. Based on a comparison between our freeboard inferred water depths and measured water depths, the median and maximum draft values produced using this method provide a robust constraint on actual water depth, with an uncertainty on inferred water depths of ~18%. However, this method was limited by the availability of stereo image pairs of stranded icebergs. To expand the spatial extent for which we inferred water depths, we calculated a characteristic depth–width ratio for each parent glacier iceberg source and inferred water depths through application of the depth–width ratio to iceberg widths from non-stereo optical satellite imagery.

To test the accuracy with which icebergs can be used to infer water depths and illustrate their utility, we applied our methods in Ilulissat Isfjord and Nujaarsuit Fjord (part of the Upernavik Fjord complex). Where measured bathymetry values were available, we compared our inferred water depths with measured water depths. Where our inferred water depths were outside the spatial extent of measurements, we regridded our data with the previously existing datasets to produce more realistic bathymetric contour maps. Overall, we found that although the uncertainties on inferred water depths may be up to 93 m (based on our combined results for all icebergs), individual uncertainties are generally <40 m. These large uncertainties make the inferred water depths unsuitable for constraining bathymetric features with high enough vertical resolution to determine the ability of warm subsurface ocean waters to reach marine glacier termini. However, the methods successfully identify shallow regions and provide useful first-order constraints on fjord water depths. These constraints on the water depths in unmapped regions contribute to the interpretation and processing of profile based datasets, provide critical information to prioritize locations where ship-based measurements are most needed, and can be used to expand existing datasets (e.g. outside of presently mapped areas, such as where glacier terminus position has changed subsequent to initial mapping efforts).

## Supplementary Material

Supplemental Material

## Figures and Tables

**Table 1.** Median iceberg depth–width ratios.

Location	Depth-Width Ratio	Source
Ilulissat Isfjord	0.45	this study
	0.40	Enderlin et al. [36]
Upernavik region	0.82	this study
	0.37	Enderlin et al. [36]
Grand Banks	0.81	Hotzel and Miller [32]
Sermilik Fjord	0.68/1.41 *	Sulak et al. [56]
Rink Isbræ	0.66/1.41 *	Sulak et al. [56]

* mean value for block/cone shaped iceberg.

**Table 2.** Statistics for method evaluation.

Linear Fit Parameters	Method	Fit Statistics	II	NJ
slope = 1 fitted intercept	freeboard	RMSE	93	66
		intercept	7	16
	depth-width	RMSE	51	73
		intercept	−72	5
	both	RMSE	82	71
		intercept	−37	9
slope = 1 intercept = 0	freeboard	RMSE	93	68
	depth-width	RMSE	88	73
	both	RMSE	90	71
