# Supplementary material for: First-Order Estimates of Coastal Bathymetry in Ilulissat and Naajarsuit Fjords, Greenland, from Remotely Sensed Iceberg Observations
Source: Remote Sens (Basel). Author manuscript; Available in PMC 2019 Dec 5. (PMC6894177; doi:10.3390/rs11080935)
Supplement: Supplemental Material [file NIHMS1056951-supplement-Supplemental_Material.zip › remotesensing-465187-final-supp/Scheicketal_remotesensing465187_rev1_supplementarymaterial.pdf]

Article

Supplementary Material: First order estimates of coastal bathymetry in Ilulissat and Naajarsuit Fjords, Greenland, from remotely-sensed iceberg observations

Jessica Scheick [1,2,‡,*], Ellyn M. Enderlin [1,2,‡], Emily Miller [2,3,‡] and Gordon Hamilton [4,5,†]

1 School of Earth and Climate Sciences, University of Maine, Orono, ME, USA
2 Climate Change Institute, University of Maine, Orono, ME, USA
3 School of Marine Sciences, University of Maine, Orono, ME, USA
4 formerly at: School of Earth and Climate Sciences, University of Maine, Orono, ME, USA
5 formerly at: Climate Change Institute, University of Maine, Orono, ME, USA
* Correspondence: jbscheick@gmail.com
† deceased
‡ Current address: 303 Bryand Global Sciences Center, University of Maine, Orono, ME, USA 04469

Version February 27, 2019 submitted to Remote Sens.

1. Methods: Qualitative Bathymetry and Study Sites

Quantifying water depth in shallow regions requires the presence of deep-drafted icebergs in shallow water and sufficient stereo image pairs to construct DEMs of icebergs. Two regions analyzed for qualitative bathymetry were not well suited for quantifying bathymetry. However, the areas of iceberg drifting, stranding, and recirculation identified indicate that the manual method provides a robust means of inferring relative bathymetry and predicting the presence or absence of a large blocking feature (i.e. sill) (Figure S1). Two systems for which qualitative bathymetry was compiled were not candidates for quantitative analysis. These systems, and the reasons preventing quantification of bathymetry, are presented here. In the Karrat Fjord system of West Greenland we focused on the fjord into which Rink Isbræ terminates. Similar to the Upernavik/Naajarsuit system, Karrat contains several shallow areas/partial sills but no distinct blocking feature that spans the fjord [1]. Here, our analysis was limited by the lack of sufficient WorldView stereo image pairs to estimate water depths in shallow regions. In Sermilik Fjord, the only site in East Greenland, icebergs are calved primarily from Helheim Glacier and the fjord showed no regions of stranding (Figure S1), as corroborated by ship-based seafloor mapping [2].

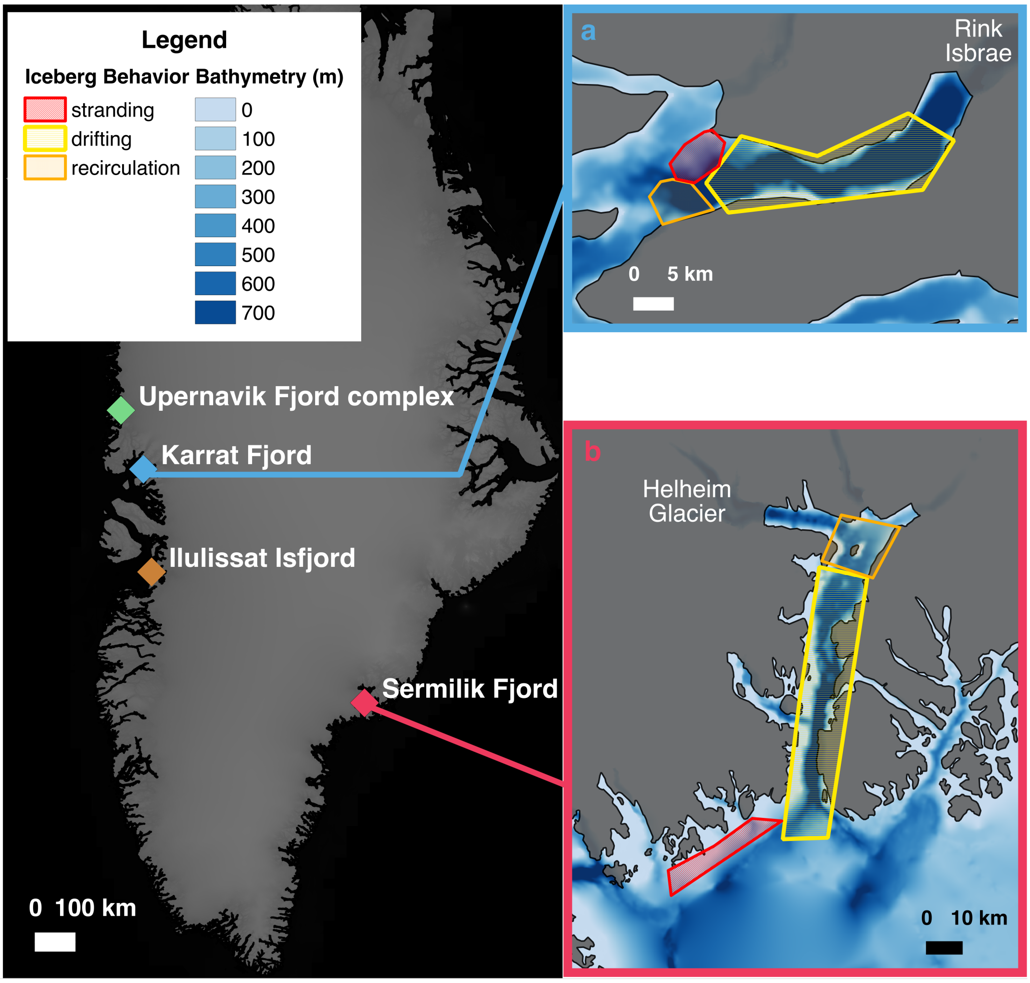

Figure 1. Qualitative bathymetry overlaid on BedMachine v3 bathymetry [3] for two locations around Greenland. Areas of stranding (red) and drifting (yellow) identified by iceberg movement correspond to bathymetric highs (light blue) and lows (dark blue), respectively. Areas with no outlines were not searched. Land is shown in grey. Glaciers supplying the majority of the icebergs in each fjord are labeled, with the location of each system identified in the panel on the left.

1. Rignot, E.; Fenty, I.; Xu, Y.; Cai, C.; Velicogna, I.; Cofaigh, C.O.; Dowdeswell, J.A.; Weinrebe, W.; Catania, G.; Duncan, D. Bathymetry data reveal glaciers vulnerable to ice-ocean interaction in Uummannaq and Vaigat glacial fjords, west Greenland. Geophys. Res. Lett. **2016**, pp. 2667–2674. doi:10.1002/(ISSN)1944-8007.
2. Straneo, F.; Hamilton, G.S.; Sutherland, D.A.; Stearns, L.A.; Davidson, F.; Hammill, M.O.; Stenson, G.B.; Rosing-Asvid, A. Rapid circulation of warm subtropical waters in a major glacial fjord in East Greenland. Nat. Geosci. **2010**, 3, 182–186. doi:10.1038/ngeo764.
3. Morlighem, M., e.a. IceBridge BedMachine Greenland, Version 3. Bathymetry., 2017. Dataset accessed [2018-06-07], doi:10.5067/2CIX82HUV88Y.

© 2019 by the authors. Submitted to Remote Sens. for possible open access publication under the terms and conditions of the Creative Commons Attribution (CC BY) license (http://creativecommons.org/licenses/by/4.0/).
