# Supplementary figures and images for: First-Order Estimates of Coastal Bathymetry in Ilulissat and Naajarsuit Fjords, Greenland, from Remotely Sensed Iceberg Observations
Source: Remote Sens (Basel). Author manuscript; Available in PMC 2019 Dec 5. (PMC6894177; doi:10.3390/rs11080935)

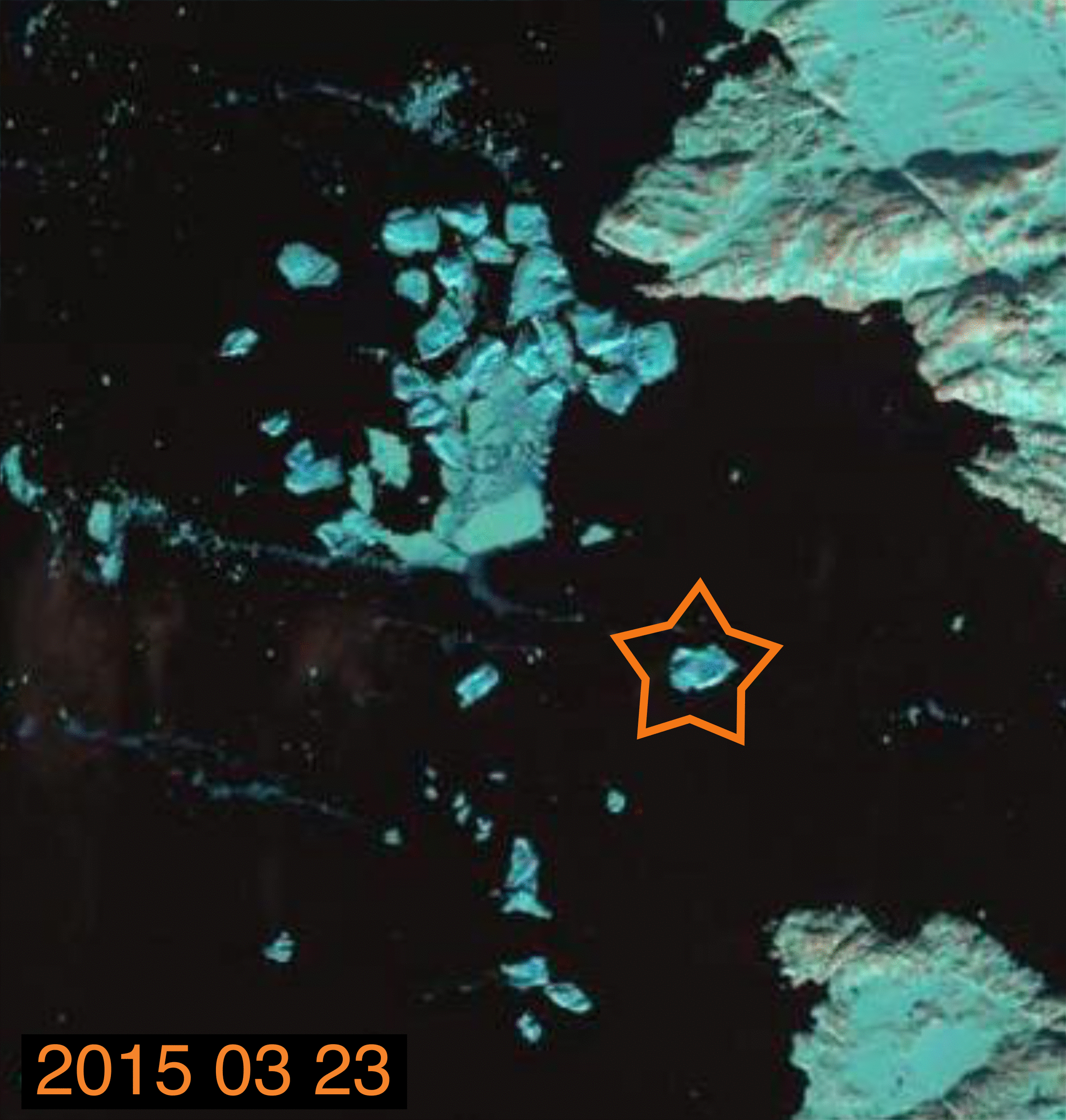

Supplement: Supplemental Material [file NIHMS1056951-supplement-Supplemental_Material.zip › remotesensing-465187-final-supp/Scheicketal_remotesensing465187_II_stranded_iceberg_movie.gif]
